# Longitudinal grey matter and metabolic contributions to cognitive changes in amyotrophic lateral sclerosis

**DOI:** 10.1093/braincomms/fcac228

**Published:** 2022-09-07

**Authors:** Thomas Hinault, Shailendra Segobin, Soumia Benbrika, Laurence Carluer, Franck Doidy, Francis Eustache, Fausto Viader, Béatrice Desgranges

**Affiliations:** Normandie University, UNICAEN, PSL Université Paris, EPHE, INSERM, U1077, CHU de Caen, GIP Cyceron, Neuropsychologie et Imagerie de la Mémoire Humaine (NIMH), Caen 14032, France; Normandie University, UNICAEN, PSL Université Paris, EPHE, INSERM, U1077, CHU de Caen, GIP Cyceron, Neuropsychologie et Imagerie de la Mémoire Humaine (NIMH), Caen 14032, France; Normandie University, UNICAEN, PSL Université Paris, EPHE, INSERM, U1077, CHU de Caen, GIP Cyceron, Neuropsychologie et Imagerie de la Mémoire Humaine (NIMH), Caen 14032, France; Normandie University, UNICAEN, PSL Université Paris, EPHE, INSERM, U1077, CHU de Caen, GIP Cyceron, Neuropsychologie et Imagerie de la Mémoire Humaine (NIMH), Caen 14032, France; Normandie University, UNICAEN, PSL Université Paris, EPHE, INSERM, U1077, CHU de Caen, GIP Cyceron, Neuropsychologie et Imagerie de la Mémoire Humaine (NIMH), Caen 14032, France; Normandie University, UNICAEN, PSL Université Paris, EPHE, INSERM, U1077, CHU de Caen, GIP Cyceron, Neuropsychologie et Imagerie de la Mémoire Humaine (NIMH), Caen 14032, France; Normandie University, UNICAEN, PSL Université Paris, EPHE, INSERM, U1077, CHU de Caen, GIP Cyceron, Neuropsychologie et Imagerie de la Mémoire Humaine (NIMH), Caen 14032, France; Normandie University, UNICAEN, PSL Université Paris, EPHE, INSERM, U1077, CHU de Caen, GIP Cyceron, Neuropsychologie et Imagerie de la Mémoire Humaine (NIMH), Caen 14032, France

**Keywords:** ALS, FDG-PET, brain atrophy, cognition, theory of mind

## Abstract

Amyotrophic lateral sclerosis is characterized by rapidly evolving cognitive and brain impairments. While previous work revealed structural and functional alterations associated with cognitive decline in patients suffering from amyotrophic lateral sclerosis, the relationships between anatomo-functional changes and both disease’s progression and the evolution of cognitive performance remain largely unexplored. Here, we took advantage of repeated multi-modal acquisitions in patients with amyotrophic lateral sclerosis over 1 year to assess the longitudinal sequence of grey matter atrophy, glucose metabolism and cognitive changes. Results revealed metabolic and structural changes over frontal, thalamic and temporal regions. Both cortical hypermetabolism and hypometabolism (right temporal gyrus and right angular gyrus, respectively) were associated with cognitive performance and thalamic hypometabolism during the follow-up testing session. Furthermore, the inferior frontal gyrus atrophy mediated the relation between early hypometabolism in this region and the subsequent decline of the theory of mind abilities. Marked volume loss was associated with larger hypometabolism and impaired cognitive performance. To our knowledge, this is the first study to longitudinally examine both grey matter volume and metabolic alteration patterns in patients with amyotrophic lateral sclerosis, over a mean follow-up time of 1 year. We identify how changes of the inferior frontal gyrus critically underly later cognitive performance, shedding new light on its high prognostic significance for amyotrophic lateral sclerosis-related changes. These results have important implications for our understanding of structural and functional changes associated with amyotrophic lateral sclerosis and how they underly cognitive impairments.

## Teaser

Cognitive impairment is frequent in patients with amyotrophic lateral sclerosis. Yet, the knowledge about longitudinal cognitive and cerebral changes in amyotrophic lateral sclerosis is scarce. Hinault *et al*. reveal (i) the association between metabolic brain changes across neuroimaging sessions and (ii) the contributions of volume and metabolic impairments to subsequent cognitive changes.

Graphical abstract

## Graphical Abstract

**Graphical abstract:**
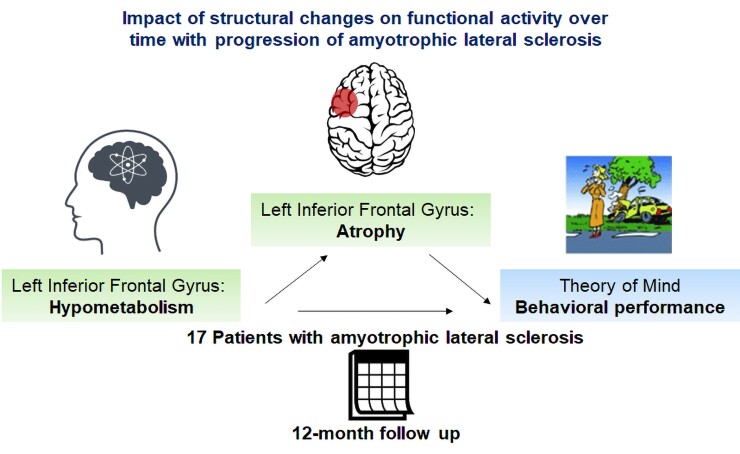

## Introduction

Amyotrophic lateral sclerosis (ALS) is the most common type of adult-onset motor neuron disease, with considerable variations in individual progression rates.^1^ Even though ALS is commonly known as a motor disorder, a growing number of studies have demonstrated the involvement of extramotor regions underlying cognition and behaviour.^2^ There is now a general recognition that ALS pathology is not restricted to a motor deficit, with about half of patients demonstrating evidence of cognitive impairments.^3^ While such cognitive decline mainly concerns executive functions,^4^ impairments of theory of mind (ToM) abilities have also been identified,^5^ which refers to the ability of understanding mental states in others. Importantly, functional adjustments can partially compensate neurological impairment in some individuals.^6^ While previous work has revealed structural and functional alterations associated with cognitive decline, the relationships between the evolution of the disease and cognitive performances remain largely unexplored.

Structural imaging mainly highlighted the atrophy of cortical^7^ and subcortical structures.^2^ Such atrophy has been associated with cognitive changes, especially in frontal regions.^8^ Among functional imaging methods, position emission tomography (PET) combined with 18F-fluorodeoxyglucose (FDG) demonstrated hypometabolism of the prefrontal cortex, fusiform gyrus and thalamus.^9^ These changes, reduced prefrontal metabolism in particular, have been associated with cognitive impairments. Hypermetabolism has also been reported relative to controls.^10^ However, these investigations of ALS-related brain and cognitive changes give no information on disease progression that can strongly vary across individuals.^11^ One critical challenge is the identification of structural and functional ALS-related brain changes that could be associated with subsequent cognitive changes and disease progression.

Longitudinal patterns of grey matter and metabolic changes may potentially give useful information on ALS progression. Longitudinal MRI studies revealed cortical thinning in frontal, temporal and parietal regions over the course of around 1 year.^12^ Recently, longitudinal atrophy of the frontal region has been associated with changes in both executive functions and ToM.^2^ However, the association with functional changes has not been assessed. In a single patient with ALS, a recent study^13^ reported the progression of hypo/hypermetabolism in the left frontal and temporal regions over the course of 20 months. To date, the respective contribution of structural and metabolic changes to individual cognitive changes associated with ALS, as well as the predictive value of initial neuroimaging findings to subsequent cognitive trajectories, remain largely unknown. Assessing this predictive value could help orient clinical decisions and improve the detection of rapidly evolving ALS patients.

Previous studies revealed important associations between the brain’s structure and function.^14,15^ However, how structural changes impact functional activity over time in the context of ALS progression remains elusive. Here, we took advantage of repeated multi-modal acquisitions in ALS patients over the course of ∼1 year to assess the longitudinal sequence of brain alterations, including grey matter (GM) atrophy, glucose hypo/hypermetabolism and cognitive changes. We considered the respective contribution of individual’s initial atrophy and metabolism and their evolution over time to better understand the variability of cognitive trajectories. Some changes can be compensatory if they are associated with preserved cognitive performance. Conversely, maladaptive changes are associated with increased cognitive decline. Results revealed both metabolic and structural changes with disease progression, especially over frontal, thalamic and temporal regions. Furthermore, cortical atrophy of the inferior frontal gyrus (IFG) was found to mediate the association between hypometabolism and cognitive functioning.

## Materials and methods

### Subjects

Seventeen patients with ALS and 30 healthy controls were included in the study (Table 1). Patients met the modified El Escorial criteria for probable or definite ALS^16^ and were diagnosed as spinal ASL. Patients were recruited via the neurology department of Caen University Hospital (France). The inclusion criteria included a non-pathological score (at least 130/144) on the Mattis Dementia Rating Scale.^17^ The exclusion criteria included the additional presence of severe and chronic illness, alcohol or drug abuse or traumatic brain injury. None of the patients fulfilled the criteria for a diagnosis of frontotemporal dementia (FTD) according to the core and supportive diagnostic features of FTD detailed in Lund and Manchester’s consensus statement.^18^ Patients underwent a neurological assessment that included the ALS Functional Rating Scale Revised (ALSFRS-R),^19^ Norris scale^20^ and Medical Research Council Muscle Strength Scale. Patients gave their written informed consent, and an independent regional ethics committee approved the study (CPP Nord-Ouest, 2008-A01150-55). They underwent a follow-up session after 9–12 months. These patients have been included in a previous structural imaging investigation.^2^ In the present study, only the 17 patients who completed both initial and follow-up neuroimaging sessions were considered in analyses. The healthy controls were recruited from the ‘*Imagerie Multimodale de la Maladie d'Alzheimer à un stade Précoce*’ (IMAP) cohort in Caen (CPP Nord-Ouest, NCT01638949). They were matched for age and gender with the ALS patients’ group and compared with patients at both sessions, as no longitudinal changes were expected in the control cohort over a period of 9–12 months. Details on psychological and cognitive assessment can be found in previous work.^2^

**Table 1:** Demographic data and cognitive data of controls and patients with ALS

	ALS patients	Controls	*P*-value
Age at baseline (years), mean ± standard deviation	61.28 ± 9.57	51.28 ± 21.73	0.079
Range	44–79	33–74	—
Male/female	9 M/8F	18 M/12F	—
Education (years)	10.26 ± 2.73	11.13 ± 3.40	0.070
Mean follow-up (months)	9.31 ± 1.03	—	—
TMT (B–A): T1	62.00	35.85	0.168
Letter verbal fluency score: T1	15.18	23.93	0.000
Episodic memory: T1	15.31	17.30	0.001
Theory of mind: T1	11.47	13.80	0.011
TMT (B–A; *N* = 12): T2	53.75		0.611
Letter verbal fluency score (*N* = 16): T2	15.14		0.000
Episodic memory (*N* = 14): T2	14		0.003
Theory of mind (*N* = 17): T2	10.82		0.009

ToM abilities were assessed with the TOM-15 task, a false-belief task which comprises 15 short comic strips.^21^ Participants had to infer each characters’ individual beliefs. Episodic memory was assessed with a task in which participants had to encode a list of 18 unrelated words.^22^ Finally, executive functions were assessed with the Trail Making Test (TMT), and a letter verbal fluency task.

### Neuroimaging data acquisition

All participants were scanned using the same MRI and PET cameras at the Cyceron centre (Caen, France): a Philipps Achieva 3.0T scanner and a discovery RX VCT 64 PET–CT device (General Electric Healthcare), respectively. For each participant, a high-resolution T_1_-weighted anatomical image was acquired using a three-dimensional fast field echo sequence (sagittal; repetition time = 20 ms, echo time = 4.6 ms, flip angle = 10°, 180 slices, slice thickness = 1 mm, field of view = 256 × 256 mm^2^, matrix = 256 × 256). Each participant underwent a PET examination the day after the MRI examination. FDG-PET scans were acquired with a resolution of 3.76 × 3.76 × 4.9 mm^3^ (field of view = 157 mm). Forty-seven planes were obtained with a voxel size of 2.7 × 2.7 × 3.27 mm^3^. Participants were fasted for at least 6 h before scanning. After a 30 min resting period in a quiet and dark environment, ≈180 MBq of FDG were intravenously injected as a bolus. A transmission scan was performed for attenuation correction and a 10 min PET acquisition scan began 50 min post-injection.

### Image processing

*MRI*: Volumetric data sets were preprocessed using the SPM12 toolbox (https://www.fil.ion.ucl.ac.uk/spm/software/spm12/). Briefly, T_1_-weighted images were spatially normalized into the Montreal Neurological Institute (MNI) space (voxel size = 1.5 mm^3^; matrix = 121 × 145 × 121) and segmented into grey matter, white matter and CSF using SPM’s unified segmentation approach, including high-velocity warp fields for the normalization procedure.^23^ The normalized grey matter and white matter images were modulated by the Jacobian determinants to preserve brain volume. The segmented images and normalization parameters estimated from this VBM protocol were used for the preprocessing of the PET data. The resulting images were smoothed by a Gaussian kernel of 8 mm full-width-at-half-maximum. The grey matter mask was obtained by averaging the unmodulated grey matter images from the healthy control group in MNI space, and thresholding the resultant mean image at 0.5.

*PET*: Data were first corrected for CSF and white matter partial volume effects in grey matter.^24^ Using SPM12, the PVE-corrected PET data set was then co-registered onto their respective native MRIs and normalized into the MNI space by reapplying the normalization parameters estimated from the VBM protocol described above (final voxel size = 2 mm3 and matrix = 79 × 95 × 79). Resultant images underwent quantitative normalization using the GM mask and were then smoothed with a Gaussian kernel of 10 mm FWHM. GM volume was quantified in the cluster showing significant differences in PET data.

### Statistical analyses

The patients at baseline and follow-up sessions were each compared with the healthy control group via *t*-tests, using age as a covariate. Each patient session was compared with the baseline metabolic and volumetric imaging of controls. The patterns of hypometabolism and hypermetabolism in patients were compared on a voxel-by-voxel basis in SPM12. Paired *t*-tests with two conditions (patients and controls) were performed to assess the pattern of GM and metabolic evolution in ALS patients during the baseline and follow-up sessions, with age entered as a covariate. T maps of all previously described analyses were thresholded using a *P* uncorrected <0.001 with a minimum cluster size of 25 voxels corresponding to a minimum regional volume of 200 mm^3^.

Correlations (age as covariate) were performed between GM volume, PET metabolism and cognitive performance to identify couplings between brain structure and activity. When both volume and metabolism of a given region of interest were correlated with the same behavioural performance (FDR corrected for multiple comparisons, the *q*-value threshold of 0.05), analyses were conducted to specify mediation relationships and to assess the respective contribution and the directionality of the relationships (following previous work^14,25^). The tested mediation model aimed to determine whether GM volume mediated the relationships between metabolism integrity and behavioural interference. The analyses estimated the direct effect (Path C′) of GM volume on behaviour after controlling for metabolism and the indirect effect (Path AB). Direct and indirect effects were computed with bias-corrected bootstrap 95% confidence intervals based on 5000 bootstrap samples.^26^ The mediation was significant if the confidence intervals in Path AB did not cross through zero. All analyses were performed using PROCESS SPSS macro for mediation analyses.^26^

### Data availability

Data sharing is not applicable to this article as no new data were created.

## Results

**Figure 1:**
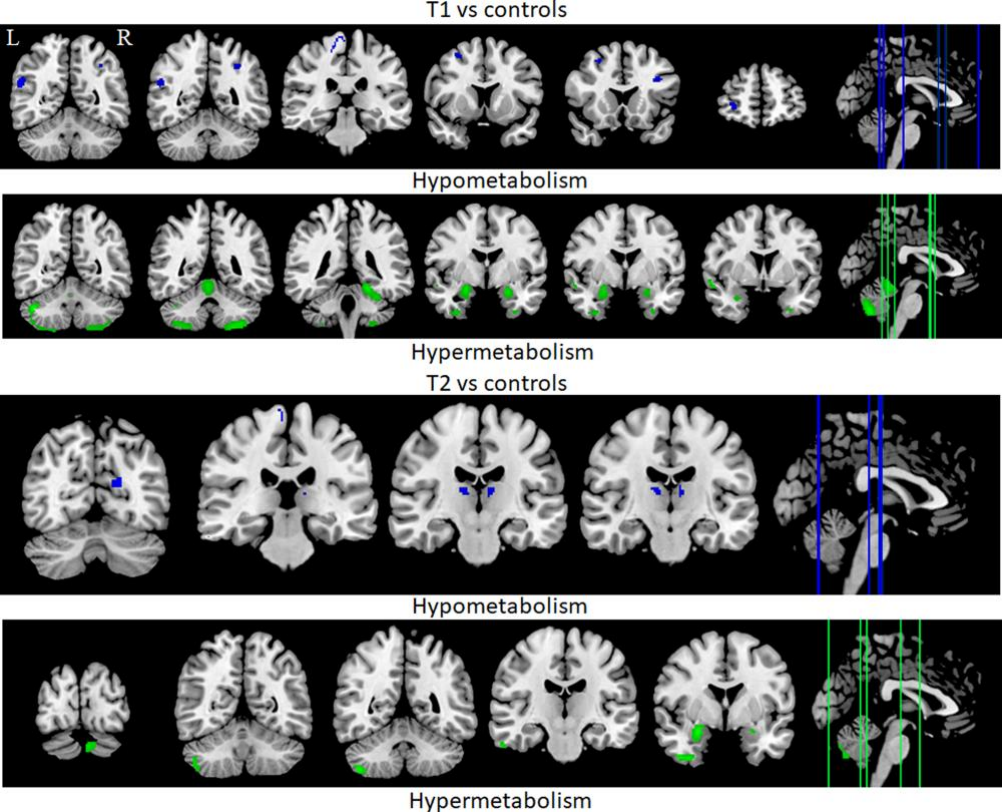
**Hypo- and hypermetabolic changes in ALS patients relative to controls during the initial (T1) and follow-up (T2) testing sessions**.

We investigated hypermetabolism and hypometabolism in ALS patients relative to controls at both T1 and T2 (see Fig. 1). No differences were observed in patients between T1 and T2. At T1, relative to controls, hypermetabolism was identified in bilateral temporal gyri, bilateral cerebellum, right amygdala and left fusiform gyrus. Hypometabolism was observed in the bilateral angular gyrus, left IFG, left ventral posterior cingulate, left primary sensory region and the right primary motor area. At T2, relative to controls, hypermetabolism was identified in the bilateral cerebellum, right inferior temporal gyrus and bilateral amygdalae. Hypometabolism was observed in the bilateral thalamic regions, left primary visual region and right primary motor regions. Regression analyses revealed associations between metabolic changes observed at T1 and T2. First, the right temporal gyrus hypermetabolism at T1 was negatively associated with the right thalamic hypometabolism at T2 (*r* = −0.727, *P* = 0.007). That is, the larger the temporal hypermetabolism, the stronger the subsequent thalamic hypometabolism. Moreover, the right angular gyrus hypometabolism at T1 was positively associated with the right thalamic hypometabolism at T2 (*r* = 0.716, *P* = 0.009). In other words, reduced metabolism of the angular gyrus was associated with reduced subsequent thalamic metabolism.

**Figure 2:**
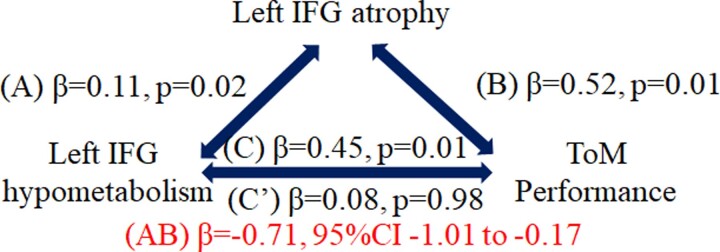
**Results of the causal mediation analysis.** The mediation involved both the IFGs grey matter volume and metabolism, and ToM performance. In the presence of significant Total effect (C) of left IFG hypometabolism on cognition, the second mediation step analysed univariate regression coefficients of left IFG structure and metabolism (Path A). This was followed by Path B analysis to determine whether IFG atrophy predicted ToM changes. Direct effects (C’; simple regressions between variables) are expressed as standardized regression coefficients. Indirect effects (AB; multiple regressions in which the predictor and the mediator are both added in the model) are expressed as partial correlation coefficients.

As our main goal was to test for potential mediation relationships of the association between brain metabolism and behavioural performance by GM volume, we identified joint correlations (FDR corrected) of cognitive performance with clusters’ hypometabolism and GM atrophy at T1 or T2, and mediation analyses were performed to specify the relationship among these variables (see Bherer *et al*.^27^ for a similar approach). That is, at T1, both hypometabolism and atrophy of the left IFG were negatively correlated with ToM performance at T2 (*r* = −0.537, *P* = 0.048 and *r* = −0.741, *P* = 0.002, respectively). No other mediation analysis was conducted, as no other region was significantly associated with a cognitive variable at both metabolic and atrophy levels. In other words, hypometabolism and atrophy at T1 were associated with reduced performance at T2. Regarding the association between PET results and GM atrophy, a significant positive correlation was identified in the left IFG (*r* = 0.722, *P* < 0.001): the larger the degree of GM atrophy, the greater the degree of hypometabolism. Mediation analyses (Fig. 2) revealed that left IFG atrophy at T1 mediated the relationship between left IFG hypometabolism at T1 and ToM performance at T2 in patients. The reversed relationship (PET hypometabolism as mediator) was not significant. Therefore, GM atrophy appears to be central in the relationship between left IFG hypometabolism and the subsequent decline of ToM abilities.

## Discussion

Approximately 50% of ALS patients have a cognitive impairment.^2^ Yet because of the difficulties associated with patients’ follow-up, the knowledge about longitudinal cognitive and cerebral changes is scarce. Here, we longitudinally investigated structural and metabolic changes and their associations with cognitive functions in non-demented ALS patients, in order to identify sensitive biomarkers of cognitive impairments. To our knowledge, this is the first study to longitudinally examine both GM volume and metabolism alteration patterns in ALS patients, over a mean follow-up time of ∼1 year. The present study (i) highlights the association between metabolic brain changes across neuroimaging sessions and (ii) shows the respective contributions of volume and metabolic impairments to subsequent cognitive changes. These results have important implications for our understanding of structural and functional changes associated with ALS and how they underly cognitive impairments.

The investigation of the association between metabolic changes at multiple time points revealed that both right temporal hypermetabolism and right AG hypermetabolism during the initial session were associated with thalamic hypometabolism during the follow-up session. Hypometabolism of the thalamus has previously been reported in ALS,^28^ together with a structural disconnection from cortical regions with disease progression.^29^ This initial hypermetabolism was associated with reduced thalamic metabolism at the follow-up session, suggesting an early disconnection preceding the reduction of thalamic activity.

The progression of ALS is associated with both compensatory and maladaptive changes. Indeed, while in some cases, the hypermetabolism of specific brain regions relative to controls has been positively associated with cognitive performance,^30^ suggesting new and/or over-recruitment to maintain efficient functioning, negative associations have also been observed. This maladaptive activity is in line with excitotoxic changes observed with the progression of neurodegenerative disease.^31^ Here, the initial temporal hypermetabolism was found to be associated with subsequent thalamic hypometabolism, which is consistent with the existing connectivity between these structures.^32^ This could reflect neurodegenerative processes and impairments of functional connectivity between temporal and thalamus regions.^33^

Although structural and functional brain changes have so far mostly been investigated separately, the complex interplay between brain metabolism and the underlying GM volume can provide a new prognostic indicator of disease progression.^34^ Previous work revealed important associations between brain’s structure and function.^14,15,35^ In ALS, progressive atrophy of the frontal region was previously shown to correlate with cognitive changes.^2^ However, how these changes interact with functional changes in the context of ALS progression remained to be further specified. Here, both IFG metabolism and GM volume during the initial testing sessions have been associated with the subsequent decline of ToM abilities. Hypometabolism levels in this region predicted GM atrophy, which in turn predicted subsequent cognitive performance. Impaired ToM is known to be a prominent feature of extramotor changes in ALS.^36^ ToM abilities correlated with the efficiency of executive processes,^37^ which are also impaired in ALS.^38^ The evolution of IFG volume in our patients is consistent with previous similar longitudinal studies of both GM atrophy and cortical thickness.^39^ Reduced IFG activity has been reported in ALS,^40^ and associated with ToM abilities.^41^ Given the implication of the IFG in higher order cognitive functions involving rule processing and updating,^42,43^ this association may reflect the processing of situations involving the use and maintenance of social rules.

Several methodological points must be considered in the interpretation of the reported findings. First, a relatively small number of participants were able to participate to this longitudinal experiment. This sample size reflects the difficulties associated with testing in this clinical population and is consistent with previous work.^44^ This patient group was also younger than controls, although the difference was not significant and age was added as covariate in statistical analyses. This could also reflect in part the fast disease progression, although the investigation of the reason for not participating in the follow-up session revealed that about half of patients invoked non-medical reasons. Moreover, earlier testing could have revealed changes preceding the reported GM atrophy. Future studies will aim at conducting longer longitudinal assessments. In summary, our longitudinal study provides several potentially important findings in the ALS field. First, results specify the pattern of brain atrophy, hypo-and hypermetabolism and their association with disease progression. Volume loss and metabolic changes over time were not only observed in the premotor cortex, but also in subcortical structures, the cerebellum, frontal and temporal regions. Second, we highlight the association between structural and functional changes with subsequent cognitive performance. We identify how IFG changes critically underly later cognitive performance, shedding new light on its high prognostic significance for ALS-related changes.

## Abbreviations

ALS =: amyotrophic lateral sclerosis
FDG =: fluorodeoxyglucose
FTP =: frontotemporal dementia
GM =: grey matter
IFG =: inferior frontal gyrus
MNI =: Montreal Neurological Institute
TMT =: Trail Making Test
ToM =: theory of mind
